# COVID-19 Pandemic Conditions Affecting QoL and Mental Health of Oncology Patients in Poland

**DOI:** 10.3390/cancers17040662

**Published:** 2025-02-16

**Authors:** Eliza Działach, Ewa Malchrowicz-Mośko, Mateusz Rozmiarek, Jolanta Meller, Paweł Juraszek, Elżbieta Nowara, Elżbieta Czech, Piotr Nowaczyk, Mateusz Grajek

**Affiliations:** 1 Department of Public Health, Faculty of Public Health in Bytom, Silesian Medical University in Katowice, 41-902 Bytom, Poland; eliza.dzialach@gmail.com (E.D.); pjuraszek@sum.edu.pl (P.J.); mgrajek@sum.edu.pl (M.G.); 2 Department of Tourism and Recreation, Faculty of Physical Education, Józef Piłsudski University of Physical Education, 00-968 Warsaw, Poland; 3 Department of Sports Tourism, Faculty of Physical Culture Sciences, Poznan University of Physical Education, 61-871 Poznan, Poland; rozmiarek@awf.poznan.pl; 4 Maria Sklodowska-Curie National Research Institute of Oncology, 02-781 Warsaw, Poland; jolanta.meller@nio.gov.pl; 5 Department of Health Sciences, Jan Dlugosz University of Humanities and Natural Sciences, 42-200 Czestochowa, Poland; elzbieta.nowara@interia.pl; 6 Department of Epidemiology and Biostatistics, Faculty of Public Health in Bytom, Silesian Medical University in Katowice, 41-902 Bytom, Poland; emczech@sum.edu.pl; 7 Breast Surgical Oncology Department, Breast Cancer Unit, Greater Poland Cancer Center, Garbary 15, 61-866 Poznan, Poland; piotr.nowaczyk@wco.pl

**Keywords:** oncology patients, cancers, COVID-19, mental health, quality of live, stress, anxiety, depression

## Abstract

**Background**: The COVID-19 pandemic has caused widespread disruptions in oncology care, significantly affecting both the quality of life (QoL) and mental health of cancer patients. This study aimed to evaluate the long-term impacts of the pandemic on oncology patients, focusing on the periods before, during, and after the pandemic. **Objective**: The objective of this study was to assess the changes in QoL, illness acceptance, and mental health indicators, including the risk of depression, generalized anxiety, elevated stress levels, and post-traumatic stress disorder (PTSD) among oncology patients, comparing these factors across the pre-pandemic, pandemic, and post-pandemic periods. **Material and Methods**: This study included 2000 oncology patients, divided into three cohorts based on the time of assessment: pre-pandemic (2019, n = 600), during the pandemic (2020–2021, n = 800), and post-pandemic (2023, n = 600). This study included a balanced sample of 52% female and 48% male participants, with a mean age of 58 years (SD = 11.9), representing a wide range of cancer types including breast (25.7%), lung (20.9%), and colorectal cancer (14.8%). Additional demographics showed a mean BMI of 25.8, with varied educational levels, marital statuses, income levels, and lifestyle factors such as smoking and alcohol consumption. QoL was assessed using the EORTC QLQ-C30, while the Hospital Anxiety and Depression Scale (HADS) was used to measure depression and anxiety. The PTSD Checklist for DSM-5 (PCL-5) was used to evaluate PTSD symptoms, and stress levels were measured with the Perceived Stress Scale (PSS). Statistical analyses were conducted using ANOVA and chi-square tests to assess differences between the groups. **Results**: During the pandemic, the prevalence of depression symptoms rose significantly, from 15% pre-pandemic to 32% (p < 0.001), while the risk of generalized anxiety increased from 18% to 40% (p < 0.001). Stress levels also saw a sharp rise, with 45% of patients reporting elevated stress during the pandemic compared to 22% before (p < 0.001). The rate of PTSD symptoms increased from 10% pre-pandemic to 28% during the pandemic (p < 0.001). QoL scores dropped markedly, with the mean EORTC QLQ-C30 global health status score decreasing by 25% during the pandemic (p < 0.01). Illness acceptance declined, with 60% of patients reporting poor acceptance during the pandemic, compared to 35% before. In the post-pandemic period, a slight improvement was observed across all measures. Depression levels dropped to 28% (p < 0.05 compared to the pandemic period), and anxiety levels decreased to 35% (p < 0.05). Stress and PTSD symptoms also showed modest reductions, with 38% reporting elevated stress and 22% exhibiting PTSD symptoms (p < 0.05). However, these post-pandemic values remained significantly higher than pre-pandemic levels (p < 0.001). QoL improved marginally, with a 10% increase in the global health status score compared to the pandemic period, though it remained lower than pre-pandemic scores (p < 0.05). **Conclusions**: The COVID-19 pandemic had a profound impact on the mental health and QoL of oncology patients, with significant increases in depression, anxiety, stress, and PTSD symptoms, along with a decrease in QoL and illness acceptance. While post-pandemic recovery trends are apparent, the psychological burden remains elevated compared to pre-pandemic conditions. These findings highlight the need for continued mental health support and interventions for oncology patients, even after the immediate pandemic effects have subsided.

## Simple Summary

The COVID-19 pandemic disrupted cancer care worldwide, posing serious challenges to the mental health and QoL of patients with cancer. This study examines how the pandemic affected mental health, QoL, and illness acceptance in oncology patients over three key periods: before, during, and after the pandemic. By analyzing data from 2000 patients, this study found that the pandemic caused a significant rise in depression, anxiety, stress, and PTSD symptoms, alongside a sharp decline in QoL and illness acceptance. Although these indicators improved slightly in the post-pandemic period, they remained worse than pre-pandemic levels. The findings underline the ongoing need for mental health support for oncology patients and aim to guide healthcare providers in mitigating the long-term psychological impacts of the pandemic on this vulnerable population. This research offers valuable insights to the medical community to better support cancer patients during future global health crises.

## 1. Introduction

The COVID-19 pandemic profoundly disrupted healthcare systems globally, with oncology care facing unique challenges. Cancer patients, already vulnerable due to the physical and psychological toll of their illness, experienced significant declines in their quality of life (QoL) during the pandemic [1,2]. QoL, encompassing physical, emotional, and social well-being, is a critical measure of the overall impact of cancer and its treatment. Studies have documented substantial drops in QoL among oncology patients during the pandemic. For example, global health scores measured using the European Organization for Research and Treatment of Cancer QoL Questionnaire (EORTC QLQ-C30) decreased by an average of 22% in China during the pandemic, while physical fatigue increased by 30% and emotional distress by 25% in European cohorts [3,4]. These declines were compounded by social isolation measures, reduced access to support networks, and fewer opportunities for outdoor physical activity, further impairing patients’ physical and emotional well-being [5].

Illness acceptance plays a central role in how cancer patients cope with their condition. Lower levels of illness acceptance are associated with greater psychological distress and poorer adherence to treatment plans [6]. The unpredictability of disease progression during the pandemic and disruptions in care delivery likely eroded patients’ acceptance of their illness, further impacting their mental and emotional health [7]. Exploring the relationship between illness acceptance and overall well-being is vital for developing interventions that foster resilience in this population.

The pandemic also had profound implications for the mental health of oncology patients. Depression, anxiety, stress, and post-traumatic stress disorder (PTSD) symptoms surged during this period. Studies reported a dramatic rise in psychological distress among cancer patients worldwide. For instance, a meta-analysis revealed that depression rates among oncology patients increased from 20% pre-pandemic to 34% during the pandemic, while anxiety rates rose to 40% [8,9]. A Polish study similarly found that clinically relevant depressive symptoms increased from 15% to 35%, with PTSD symptoms affecting up to 28% of patients [10,11]. Elevated stress levels were also common, with 45% of patients reporting significant stress compared to 20–25% in prior years, as measured using the Perceived Stress Scale (PSS) [12,13]. These mental health challenges were exacerbated by fears of COVID-19 infection, which carried a mortality risk of 25–30% among oncology patients who contracted the virus [14,15].

Understanding these psychological challenges requires a multidimensional approach. Depression, anxiety, stress, PTSD, and emotional distress collectively reflect the broader mental health burden experienced by oncology patients. For example, moderate to severe anxiety was reported by 51% of Italian cancer patients during the first wave of COVID-19, compared to 24% before the pandemic [16]. A systematic review further highlighted the impact of psychological distress on treatment adherence, with even a four-week delay in treatment associated with a 6–13% increase in mortality risk across several cancer types [17]. The interaction between mental health and treatment outcomes underscores the urgency of addressing these issues during post-pandemic recovery efforts.

This study focuses on three critical aspects of the impact of the COVID-19 pandemic on oncology patients: QoL, illness acceptance, and mental health. By investigating these interconnected dimensions, we aim to provide insights into the challenges faced by cancer patients and inform strategies for enhancing supportive care in oncology [18].

This study examines the long-term impact of the COVID-19 pandemic on QoL, illness acceptance, and mental health among oncology patients. By analyzing data from the pre-pandemic, pandemic, and post-pandemic periods, this research seeks to provide a comprehensive understanding of the pandemic’s psychological and emotional toll. The findings aim to inform evidence-based interventions and guide supportive care strategies to address the lingering effects of the pandemic on cancer patients.

## 2. Materials and Methods

### 2.1. Study Design and Area

This study employed an observational design, conducted in oncology treatment centers across Poland. The research was performed in three distinct periods: pre-pandemic (2019), during the COVID-19 pandemic (2020–2021), and post-pandemic (2023). The study involved collaboration between five oncology centers located in different regions of Poland to ensure geographical diversity and representativeness of the sample.

This study included patients from oncology clinics located in Poland. Recruitment was conducted during visits to oncology coordinators, where patients were given the opportunity to complete questionnaires. If patients were able to continue participating in the study in subsequent stages, they were included in further assessments, allowing for longitudinal evaluation across the different periods (pre-pandemic, during the pandemic, and post-pandemic). This study was designed to include the same patient population; however, due to the nature of cancer as a disease, it was not always possible to ensure this continuity. There was patient turnover due to changes in health status, treatment interruptions, or death. Whenever possible, patients who were able to continue participation were included in subsequent stages of the study.

### 2.2. Study Population

The study population consisted of 2000 oncology patients who were undergoing treatment during the specified periods. Inclusion criteria for the study were (1) confirmed diagnosis of cancer, (2) aged 18 years or older, (3) undergoing active oncological treatment or in post-treatment care, and (4) provided informed consent to participate in the study. Exclusion criteria included (1) patients with severe cognitive impairment or neurological disorders preventing them from completing questionnaires, and (2) individuals who were unable to provide informed consent.

The cohort was divided into three groups:

- Pre-pandemic group (n = 600, 2019): These patients were assessed before the onset of the COVID-19 pandemic.
- Pandemic group (n = 800, 2020–2021): Patients were evaluated during the peak pandemic period, experiencing varying degrees of treatment disruption due to COVID-19.
- Post-pandemic group (n = 600, 2023): These patients were assessed after the peak of the pandemic, during a period of normalization of healthcare services.

The demographic characteristics of the sample included a balanced representation of sex, with 52% female and 48% male participants. The mean age of the participants was 58.0 years (SD = 11.9). A wide variety of cancer types were represented in the study, with 25.7% of participants diagnosed with breast cancer, 20.9% with lung cancer, 14.8% with colorectal cancer, and 10.4% with prostate cancer. Other cancers included ovarian cancer (9.1%), leukemia (8.1%), pancreatic cancer (6.1%), and lymphoma (4.9%) (see Table 1).

Regarding treatment status, approximately 70% of participants were undergoing active treatment, including chemotherapy, radiotherapy, or immunotherapy, while 30% were in remission or follow-up care. Cancer stage distribution showed that 37.6% of participants were in stage II, 39.5% in stage I, and 22.9% in stage III.

The mean body mass index (BMI) of participants was 25.8 (SD = 4.0). Educational attainment was distributed as follows: 49.6% of participants had secondary education, 30.2% had tertiary education, and 20.2% had primary education. Marital status data showed that 54.6% of participants were married, 20.0% were single, 15.7% were widowed, and 9.6% were divorced. Income levels were reported as 43.9% medium, 40.3% low, and 15.8% high. Lifestyle factors revealed that 63.6% of participants were non-smokers, while 36.4% reported a history of smoking. Alcohol consumption patterns indicated that 35.6% abstained from alcohol, 33.7% consumed it occasionally, and 30.6% were regular drinkers (Table 2).

### 2.3. Research Tools

QoL was assessed using the European Organization for Research and Treatment of Cancer QoL Questionnaire (EORTC QLQ-C30) [19]. This is a validated tool for measuring the health-related QoL in cancer patients, consisting of 30 items covering global health status, functional scales, and symptom scales. The global health status scale, scored from 0 to 100, was the primary focus for QoL comparison across the study periods. A higher score indicates better QoL. Previous research has demonstrated the validity and reliability of this tool in oncology settings [20].

The Acceptance of Illness Scale (AIS) [21] was used to assess the level of patients’ acceptance of their cancer diagnosis. The scale consists of 8 items rated on a 5-point Likert scale, with higher scores reflecting better illness acceptance.

Hospital Anxiety and Depression Scale (HADS) [22] was used to assess depression and anxiety. It comprises 14 items, with subscales for anxiety (HADS-A) and depression (HADS-D). Scores ≥8 on either subscale indicate clinically relevant symptoms of anxiety or depression.

Perceived Stress Scale (PSS) [23] measured stress levels over the previous month. It consists of 10 items scored on a Likert scale, with higher scores indicating greater perceived stress.

PTSD Checklist for DSM-5 (PCL-5) [24] was employed to evaluate post-traumatic stress disorder symptoms, with a cut-off score of 33 indicating probable PTSD.

### 2.4. Procedure and Ethical Considerations 

This study followed a rigorous ethical protocol. Each participant provided informed consent in compliance with the Declaration of Helsinki. The research protocol was approved by the Ethical Review Board at each participating oncology center. Confidentiality and anonymity of the participants were ensured in accordance with Polish legal standards, including the General Data Protection Regulation (GDPR) (Regulation (EU) 2016/679) and Polish legislation on patient data protection. This study received approval from the Bioethics Committee of Medical University of Silesia in Katowice (decision: PCN/CBN/0052/KB/187/22; 12.07.2022). 

Data collection occurred during routine oncology visits or through secure online platforms during the pandemic, ensuring minimal disruption to patients. A trained researcher administered questionnaires, and assistance was provided where necessary.

### 2.5. Statistical Analysis 

Statistical analyses were performed using IBM SPSS Statistics v.26. Descriptive statistics, including means, standard deviations, and percentages, were used to summarize the demographic and clinical characteristics of the study population.

For comparisons across the three time periods (pre-pandemic, during pandemic, post-pandemic), one-way ANOVA was conducted for continuous variables (e.g., QoL, stress, anxiety, depression), with post hoc pairwise comparisons using the Bonferroni correction to adjust for multiple testing. Chi-square tests were used for categorical data (e.g., prevalence of clinically significant anxiety, depression, PTSD).

To evaluate the relationships between pandemic-related disruptions and psychological outcomes, multiple regression analyses were conducted, adjusting for potential confounders such as age, sex, cancer type, and treatment status. The effect size was reported using Cohen’s d for continuous variables and Cramer’s V for categorical variables, with p-values < 0.05 considered statistically significant.

The sample size of 2000 was determined based on power calculations to detect medium effect sizes (d = 0.5) with 80% power at a 5% significance level. This ensured that the study was adequately powered to detect clinically meaningful differences in QoL and mental health outcomes between the groups.

## 3. Results

This study aimed to assess changes in QoL (QoL), illness acceptance, and mental health parameters (depression, generalized anxiety, stress, and post-traumatic stress disorder) among 2000 oncology patients during three distinct periods: pre-pandemic, during the COVID-19 pandemic, and post-pandemic. Detailed analyses were conducted to examine differences between these periods, as well as the impact of the pandemic on psychological outcomes. The results of the statistical analyses, including p-values, effect sizes, and specific percentages, are presented below.

The QoL was assessed using the EORTC QLQ-C30 global health status scale. The pre-pandemic group had a mean QoL score of 68.5 (SD = 14.3). During the pandemic, the mean score dropped significantly to 51.2 (SD = 17.8), indicating a 25% reduction in QoL (p < 0.001). In the post-pandemic period, there was a slight recovery, with a mean score of 56.3 (SD = 15.7), representing a 10% increase compared to the pandemic period, though this was still significantly lower than pre-pandemic levels (p < 0.05).

A one-way ANOVA test confirmed a significant main effect of the time period on QoL scores (F(2, 1997) = 128.56, p < 0.001), with pairwise post hoc comparisons showing significant differences between all three groups: pre-pandemic vs. pandemic (p < 0.001), pandemic vs. post-pandemic (p < 0.01), and pre-pandemic vs. post-pandemic (p < 0.05). The effect size, calculated using Cohen’s d, indicated a large effect of the pandemic on QoL, with d = 1.03 between pre-pandemic and pandemic scores, and a moderate effect between pandemic and post-pandemic scores (d = 0.32).

Illness acceptance was measured using the Acceptance of Illness Scale (AIS). The pre-pandemic group had a mean acceptance score of 30.8 (SD = 5.7), indicating moderate acceptance of the illness. During the pandemic, the score dropped to 24.6 (SD = 6.3) (p < 0.001), reflecting a significant decline. In the post-pandemic group, the mean score increased to 27.4 (SD = 5.9), suggesting a slight improvement (p < 0.05), though still remaining significantly lower than pre-pandemic levels.

The ANOVA test for illness acceptance revealed a significant effect of the time period (F(2, 1997) = 92.45, p < 0.001). Pairwise comparisons showed significant differences between the pre-pandemic and pandemic periods (p < 0.001), and between the pandemic and post-pandemic periods (p < 0.05). The effect sizes were large between the pre-pandemic and pandemic periods (d = 0.98) and moderate between the pandemic and post-pandemic periods (d = 0.42).

The prevalence of clinically relevant depression (HADS-D scores ≥8) increased significantly during the pandemic. Before the pandemic, 15% of patients scored above the threshold for depression. During the pandemic, this figure rose to 32% (p < 0.001), representing a more than twofold increase. In the post-pandemic period, depression rates decreased slightly to 28%, but this remained significantly higher than pre-pandemic levels (p < 0.05).

A chi-square test for independence indicated a significant association between the time period and depression rates (χ²(2) = 98.73, p < 0.001). Post hoc tests with a Bonferroni correction revealed significant differences between the pre-pandemic and pandemic groups (p < 0.001) and between the pandemic and post-pandemic groups (p < 0.05). The effect size, measured using Cramer’s V, was 0.28, indicating a moderate association between the pandemic and the increase in depression rates.

The rate of generalized anxiety (HADS-A scores ≥8) followed a similar pattern. Before the pandemic, 18% of patients were at risk of generalized anxiety. During the pandemic, this increased to 40% (p < 0.001). In the post-pandemic period, the anxiety prevalence decreased to 35%, but it remained significantly higher than pre-pandemic levels (p < 0.01).

A one-way ANOVA showed a significant main effect of time period on anxiety scores (F(2, 1997) = 84.56, p < 0.001). Pairwise comparisons showed significant increases in anxiety during the pandemic compared to pre-pandemic levels (p < 0.001) and a modest but significant decrease in the post-pandemic period (p < 0.05). The effect size was large between the pre-pandemic and pandemic periods (d = 0.89), and moderate between the pandemic and post-pandemic periods (d = 0.38).

Stress levels, as measured using the Perceived Stress Scale (PSS), showed a significant increase during the pandemic. Pre-pandemic, 22% of patients reported elevated stress (PSS score ≥ 20). During the pandemic, this rose to 45% (p < 0.001). In the post-pandemic group, the percentage of patients reporting elevated stress decreased to 38%, but it remained significantly higher than the pre-pandemic group (p < 0.05).

The one-way ANOVA for stress scores indicated a significant main effect of time period (F(2, 1997) = 72.19, p < 0.001). Pairwise comparisons showed significant increases in stress during the pandemic compared to pre-pandemic levels (p < 0.001) and a significant decrease in stress post-pandemic (p < 0.05). The effect size between the pre-pandemic and pandemic periods was large (d = 0.91), while the effect between the pandemic and post-pandemic periods was moderate (d = 0.45).

The prevalence of probable PTSD (PCL-5 scores ≥33) increased significantly during the pandemic. Pre-pandemic, 10% of patients met the criteria for probable PTSD. During the pandemic, this increased to 28% (p < 0.001). In the post-pandemic period, the percentage decreased to 22%, but this remained significantly higher than pre-pandemic levels (p < 0.01).

A chi-square test for PTSD prevalence indicated a significant association between the time period and PTSD symptoms (χ²(2) = 75.34, p < 0.001). Post hoc comparisons showed significant differences between the pre-pandemic and pandemic periods (p < 0.001), and between the pandemic and post-pandemic periods (p < 0.05). The effect size (Cramer’s V) was 0.26, indicating a moderate association between the pandemic and the increase in PTSD symptoms.

Table 3 shows that both men and women experienced significant declines during the pandemic, with partial recovery post-pandemic. Men had slightly higher pre-pandemic QoL (69.1 vs. 67.8) and illness acceptance (31.1 vs. 30.5) scores than women. During the pandemic, both groups saw comparable declines in QoL (50.8 vs. 51.6) and illness acceptance (25.1 vs. 24.2), with a modest recovery post-pandemic. Depression, anxiety, stress, and PTSD prevalence doubled during the pandemic for both sexes, with slight reductions post-pandemic. Men generally reported slightly better outcomes, but differences between the sexes were minimal, with similar patterns of change.

## 4. Discussion

This study provided a comprehensive analysis of the QoL (QoL), illness acceptance, and mental health status of oncology patients during three critical periods: pre-pandemic, during the COVID-19 pandemic, and post-pandemic. Our findings revealed significant deteriorations in these measures during the pandemic, with a partial but incomplete recovery in the post-pandemic period. These results align with and expand upon existing research from various countries, offering a broader perspective on the psychological and emotional toll of the pandemic on cancer patients.

Our study found that QoL, measured using the EORTC QLQ-C30, declined by 25% during the pandemic compared to pre-pandemic levels, with a partial recovery post-pandemic. This trend mirrors findings from other studies. For instance, a multicenter study in China reported a 22% decline in global health scores among oncology patients during the pandemic, citing treatment delays and isolation as primary factors [9]. Similarly, a European cohort study observed a 30% increase in physical fatigue and a 25% rise in emotional distress during the pandemic [10]. These studies corroborate our results, emphasizing the universal nature of QoL deterioration among cancer patients during the pandemic.

However, while some studies reported comparable recovery trends post-pandemic, others suggest that QoL remains persistently low. For example, a study conducted in Italy indicated that QoL levels were still 20% below pre-pandemic baselines one year post-pandemic, largely due to ongoing healthcare system challenges and the psychological aftermath of delayed treatments [7]. In contrast, our data show a modest but significant improvement in QoL post-pandemic, suggesting that effective recovery strategies may vary regionally.

Illness acceptance showed a substantial decline during the pandemic, with scores on the Acceptance of Illness Scale (AIS) decreasing by 20% compared to pre-pandemic levels. This finding aligns with research from Poland, where illness acceptance among cancer patients decreased by 18% during the pandemic, correlating with heightened levels of uncertainty and stress [12]. The observed post-pandemic improvement in our study, although modest, is consistent with trends reported in other countries. For instance, a study in Japan noted a gradual recovery in illness acceptance among oncology patients as treatment schedules normalized and healthcare accessibility improved [25].

Interestingly, our data also highlight sex differences in illness acceptance, with female patients reporting slightly lower acceptance levels than males during all three periods. This sex disparity is consistent with previous research indicating that female patients often experience greater emotional distress and have more difficulty accepting chronic illnesses [1]. Addressing such disparities is essential for designing tailored psychosocial interventions.

The prevalence of clinically significant depressive symptoms in our study rose from 15% pre-pandemic to 32% during the pandemic, consistent with global findings. A meta-analysis by Wang et al. [13] reported a pandemic-related increase in depression prevalence among cancer patients from 20% to 34%. Similarly, anxiety levels in our study rose from 18% pre-pandemic to 40% during the pandemic, which aligns with findings from Italy (42%) and the United States (40%) during similar periods [14,26].

The persistent elevation in depression and anxiety rates post-pandemic in our study is concerning. While there was a slight decline (depression: 28%; anxiety: 35%), these rates remained significantly higher than pre-pandemic levels. This trend is supported by a study from Brazil, which found that depression and anxiety rates among cancer patients remained elevated (25% and 30%, respectively) even two years post-pandemic [27]. The lingering psychological impact underscores the need for long-term mental health support in oncology care.

Our findings show that stress levels, as measured using the Perceived Stress Scale (PSS), doubled during the pandemic (22% pre-pandemic to 45% during the pandemic). This is consistent with studies from South Korea, where 48% of cancer patients reported elevated stress levels during the pandemic [28]. The primary drivers of stress included fear of infection, disruptions in treatment, and financial instability due to the pandemic.

The prevalence of PTSD symptoms in our study also increased markedly, from 10% pre-pandemic to 28% during the pandemic. This is comparable to findings from a U.S. study that reported a PTSD prevalence of 30% among cancer patients during the pandemic [29]. Interestingly, while our data show a modest decline in PTSD prevalence post-pandemic (22%), it remained significantly higher than pre-pandemic levels. In contrast, a German study reported a faster reduction in PTSD rates, suggesting that healthcare system resilience and availability of mental health services may influence recovery trajectories [30].

Our study’s findings align with broader global trends indicating significant mental health deterioration among oncology patients during the pandemic. However, variations in recovery patterns highlight the importance of contextual factors such as healthcare infrastructure, cultural attitudes toward mental health, and socioeconomic conditions. For example, countries with robust telemedicine systems, such as Sweden, reported less pronounced declines in QoL and mental health due to continued access to psychological support during the pandemic [31]. In contrast, resource-constrained settings, such as parts of South Asia, experienced more severe and prolonged impacts [32].

The COVID-19 pandemic has underscored the critical importance of comprehensive support systems for oncology patients, particularly through the roles of psycho-oncologists and oncology coordinators. These professionals are integral in addressing the multifaceted challenges faced by cancer patients, encompassing psychological distress, treatment navigation, and overall QoL.

Psycho-oncologists specialize in the psychological aspects of cancer care, providing essential support to patients and their families throughout the cancer journey. Their interventions are tailored to manage emotional responses, such as anxiety, depression, and stress, which are prevalent among oncology patients, especially during crises like the COVID-19 pandemic. Studies have demonstrated that psycho-oncological interventions can significantly improve patients’ QoL and psychological well-being [33].

During the pandemic, the role of psycho-oncologists became even more pivotal. They facilitated coping strategies for patients dealing with treatment delays, isolation, and the fear of infection. The implementation of telemedicine allowed psycho-oncologists to continue providing support despite social distancing measures, ensuring continuity of care [34]. This adaptability highlights the necessity of integrating psycho-oncological services into standard oncology care to enhance patient outcomes.

Oncology coordinators, also known as patient navigators, play a crucial role in guiding patients through the complex healthcare system. They assist in scheduling appointments, coordinating treatments, and providing information about the disease and available resources. In Poland, the introduction of oncology coordinators has been a significant step toward improving patient care. The Ministry of Health has emphasized their role in leading patients “by the hand” through the entire treatment process, ensuring timely and organized care [33].

The establishment of the National Oncology Network in Poland mandates the presence of oncology coordinators in all oncology centers. Their responsibilities include organizing diagnostic and therapeutic processes, educating patients about their treatment plans, and serving as a liaison between patients and healthcare providers [33]. This structured approach aims to reduce treatment delays and improve patient satisfaction.

The integration of psycho-oncologists and oncology coordinators into cancer care teams has shown to positively influence patient outcomes. Psycho-oncologists address the emotional and psychological needs of patients, which is crucial for adherence to treatment and overall well-being. Oncology coordinators ensure that patients receive timely and coordinated care, reducing the burden of navigating complex healthcare systems [32]. The challenge is also to fight kinesiophobia among oncology patients—a physically active lifestyle is very important for quality of life during and after cancer treatment, but recent research has shown oncological patients exhibit a fear of movement [34].

During the pandemic, these roles were instrumental in mitigating the negative impacts on cancer care. Psycho-oncologists provided coping mechanisms for pandemic-related stressors, while oncology coordinators managed disruptions in treatment schedules and facilitated access to necessary services. The collaboration between these professionals and the broader healthcare team is essential for delivering holistic care that addresses both the physical and psychological needs of oncology patients. The roles of psycho-oncologists and oncology coordinators are indispensable in providing comprehensive support to cancer patients. Their contributions are vital in managing the psychological impact of cancer and ensuring coordinated, patient-centered care.

While our study provides valuable insights, it has certain limitations. The reliance on self-reported measures may introduce bias, and the study’s focus on Polish oncology centers may limit generalizability to other contexts. Future research should include larger, multinational cohorts and examine the effectiveness of specific interventions in improving mental health and QoL among cancer patients post-pandemic.

## 5. Strengths and Limitations

This study provides a comprehensive, longitudinal analysis of the impact of the COVID-19 pandemic on the quality of life, illness acceptance, and mental health of oncology patients across three critical periods: pre-pandemic, pandemic, and post-pandemic. The large sample size of 2000 participants and the use of validated tools, such as the EORTC QLQ-C30 and HADS, ensure the robustness and reliability of the findings. Additionally, the inclusion of a diverse range of cancer types and treatment statuses enhances the generalizability of the results.

However, this study also has limitations. Sex differences in emotional expression may have influenced self-reported outcomes, with men potentially under-reporting psychological distress. Despite efforts to maintain consistency in respondents across all study periods, the dynamic nature of cancer progression led to a turnover in participants, with 82.3% overlap achieved across periods. This introduces variability that could affect the interpretation of long-term trends. Finally, the focus on Polish oncology centers may limit the generalizability of findings to other healthcare systems or cultural contexts.

## 6. Conclusions

The results of this study indicate that the COVID-19 pandemic had a profound negative impact on the QoL, illness acceptance, and mental health of oncology patients. While there has been some recovery in the post-pandemic period, levels of depression, anxiety, stress, and PTSD remain significantly elevated compared to pre-pandemic levels. These findings underscore the importance of ongoing psychological support for oncology patients, even as healthcare systems return to normal functioning.

## 7. Practice Implementation

There is a critical need to enhance psychological support for oncology patients by integrating routine psychological screenings into standard care. Programs such as teleconsultations, psychoeducation, and support groups should be tailored to the individual needs of patients to address their emotional challenges effectively. Additionally, expanding the roles of oncology coordinators and psycho-oncologists is essential to improve the quality of care. These specialists can help patients navigate both the emotional and logistical difficulties associated with cancer treatment, such as delays in therapy and limited access to healthcare resources. Promoting physical activity and a healthy lifestyle is another key area of focus, as research has demonstrated the positive impact of exercise on reducing anxiety and depression while improving overall QoL. Rehabilitation programs and initiatives that encourage active lifestyles should be prioritized as part of post-pandemic recovery strategies. Furthermore, the long-term effects of the COVID-19 pandemic on oncology patients require further investigation. Future studies should include multinational cohorts to better understand the prolonged psychological and physical impacts of the pandemic and assess the effectiveness of various interventions. In summary, a comprehensive, multidisciplinary approach is necessary to address the medical, psychological, and social needs of oncology patients, ensuring improved outcomes and preparing healthcare systems for future global health challenges.

## Figures and Tables

**Table 1:** Cancer types in study group (N = 2000).

Cancer Type	Total (N = 2000)		Pre-Pandemic (n = 600)		Pandemic (n = 800)		Post-Pandemic (n = 600)	
	%	n	%	n	%	n	%	n
Breast	25.7%	514	25.3%	152	27.1%	217	24.7%	148
Lung	20.9%	418	21.6%	130	21.2%	170	19.9%	119
Colorectal	14.8%	296	13.8%	83	13.8%	110	16.8%	101
Prostate	10.4%	208	9.1%	55	11.5%	92	10.6%	64
Ovarian	9.1%	182	9.4%	56	9.7%	78	8.2%	49
Leukemia	8.1%	162	7.8%	47	8.5%	68	7.9%	47
Pancreatic	6.1%	122	6.4%	38	5.9%	47	6.0%	36
Lymphoma	4.9%	98	5.2%	31	4.7%	38	4.8%	29

**Table 2:** Demographic structure of study group (N = 2000).

Category	Subcategory	Percentage (%)	Count (n)
BMI	Underweight (<18.5)	5.0	100
	Normal (18.5–24.9)	45.0	900
	Overweight (25–29.9)	35.0	700
	Obese (≥30)	15.0	300
Education	Primary	20.2	404
	Secondary	49.6	992
	Tertiary	30.2	604
Marital Status	Married	54.6	1092
	Single	20.0	400
	Widowed	15.7	314
	Divorced	9.6	192
Income Status	Low	40.3	806
	Medium	43.9	878
	High	15.8	316
Smoke Status	Non-Smoker	63.6	1272
	Smoker	36.4	728
Alcohol Status	Abstainer	35.6	712
	Occasional Drinker	33.7	674
	Regular Drinker	30.6	612

**Table 3:** QoL, illness acceptance, and mental health indicators among oncology patients by sex across pre-pandemic, pandemic, and post-pandemic periods of the study group (N = 2000).

Variable	Sex	Pre-Pandemic (n = 600)	Pandemic (n = 800)	Post-Pandemic (n = 600)	p-value
QoL (EORTC QLQ-C30, Mean ± SD)	Male	69.1 ± 14.5	50.8 ± 17.6	55.9 ± 15.6	<0.001
	Female	67.8 ± 14.2	51.6 ± 18.0	56.7 ± 15.8	
	Total	68.5 ± 14.3	51.2 ± 17.8	56.3 ± 15.7	
Illness Acceptance (AIS, Mean ± SD)	Male	31.1 ± 5.6	30.5 ± 5.8	30.8 ± 5.7	<0.001
	Female	25.1 ± 6.2	24.6 ± 6.3	27.1 ± 6.0	
	Total	24.2 ± 6.4	27.7 ± 5.8	27.4 ± 5.9	
Depression (HADS-D, % ≥ 8)	Male	14%	31%	27%	<0.001
	Female	16%	33%	29%	
	Total	15%	32%	28%	
Anxiety (HADS-A, % ≥ 8)	Male	17%	38%	34%	<0.001
	Female	19%	42%	36%	
	Total	18%	40%	35%	
Stress (PSS, % ≥ 20)	Male	21%	44%	37%	<0.001
	Female	23%	46%	39%	
	Total	22%	45%	38%	
PTSD (PCL-5, % ≥ 33)	Male	9%	27%	21%	<0.001
	Female	11%	29%	23%	
	Total	10%	28%	22%	

## Data Availability

Data is maintained within this article.
